# How boots affect the kinematics and kinetics of lower limb joints during walking compared to casual footwear

**DOI:** 10.1038/s41598-024-68533-1

**Published:** 2024-08-08

**Authors:** Farzaneh Haghighat, Mohammadreza Rezaie, Mahdi Majlesi

**Affiliations:** 1 Rehabilitation Sciences Research Center, Shiraz University of Medical Sciences, Shiraz, Iran; 2 Independent Researcher, Shiraz, Iran; 3 Department of Sport Biomechanics, Hamedan Branch, Islamic Azad University, Hamedan, Iran

**Keywords:** Biomedical engineering, Health sciences

## Abstract

Boots are widely used by many people for various purposes, but their impact on gait biomechanics and injury risk is not well understood. This study investigated the effects of boots on walking biomechanics, compared to casual footwear. The lower limb joint kinematics and kinetics of 20 healthy male participants aged 20 to 30 years old were compared during self-paced walking with boots and shoes. The results showed that walking with boots is associated with greater hip extensor (P = 0.009) and ankle dorsiflexor (P < 0.001) moments in early stance, hip power generation (P < 0.001) and knee power absorption (P < 0.001) in early swing phase, hip abductor (P < 0.001) and knee adduction (P < 0.001) moments in the entire stance, net concentric work for the hip joint in sagittal (13.9%, P = 0.001) and frontal (21.7%, P = 0.002) planes. In contrast, the subtalar supinator moment in the entire stance (P < 0.001), ankle angular velocity in late stance (P < 0.001), and net concentric (− 42.7%, P < 0.001) and eccentric (− 44.6%, P = 0.004) works of subtalar joint were significantly lower in the boot condition. The compensatory adjustments in the hip and knee joints may result from ankle restrictions. While boots may aid those with ankle disorders, lower limb loading and the risk of musculoskeletal injuries and osteoarthritis could be increased. This study offers new perspectives on the biomechanical impact of boots on gait, potential prevention and treatment strategies of related injuries, and advancing footwear design.

## Introduction

Footwear is an essential part of human culture and civilization. It has been used for thousands of years by people in different regions of the world for various purposes and functions. Footwear not only reflects the social, economic and environmental factors that shape human societies, but also influences the physical, psychological and emotional aspects of human life^1^. A common type of footwear is the boot, defined as “*any sort of footwear that extends proximal to the ankle*”^2^ and designed to meet a variety of activities and careers requirements.

Boots are so popular among people all around the world and frequently utilized in sports and occupational settings, such as military, firefighting, hiking, and other outdoor activities. They are designed to protect and stabilize the foot and ankle and also, to keep them safe against rugged terrain and harsh environmental conditions^3^. They also have enormous implications in clinical settings for the management of musculoskeletal disorders such as adult acquired flatfoot, ankle instability, flexible flatfoot, and clubfoot deformity^2^. They are also used for embedding the assistive devices like leg braces and partial foot prostheses in Chopart amputation in Orthotics and Prosthetics practice^2^. Although sufficient ankle range of motion (RoM) is essential for effective gait, its excessive motion is potentially detrimental and normally prevented by secondary anatomical structures, such as the muscles and ligaments. Boots effectively reduce the incidence of ankle strain/sprain injuries by reinforcing these anatomical structures and preventing excessive ankle motions^4,5^.

Various boot features, such as material, mass, shaft height, shaft stiffness, and sole flexibility have the potential to alter lower limb gait kinematics and kinetics, energy expenditure, muscle activity, posture, balance, and foot comfort^3,6^. Wearing boots could influence the loads on muscles, fatigue rate, energy expenditure, and consequently, risk of injury^3,7^. The literature on footwear science has extensively investigated how different types of footwear affect the performance of different tasks. Despite the numerous studies that have been done in this field, not too many have specifically investigated the impact of boots on joint biomechanics.

Previous studies have suggested that running with boots is associated with higher impact loading and greater stress on the Achilles tendon and patellofemoral joint compared to regular running shoes. Additionally, it leads to increased tibial rotation, ankle eversion, and restricted ankle RoM, which are identified as risk factors for developing lower limb musculoskeletal overuse injuries, such as iliotibial band syndrome, patellofemoral pain, tibial stress syndrome, Achilles tendon pathology, and plantar fasciitis^3^. Given that in many situations people use boots during their activities of daily living and not running, it is important to understand how boots affect the biomechanics of other activities as well.

To the best of our knowledge, only a few studies have evaluated the effects of boots on walking biomechanics in healthy individuals. EMG studies have found that walking with boots is associated with decreased activation of peroneus longus, as well as increased activation of tibialis anterior and rectus femoris^8^, vastus medialis^9^, biceps femoris and vastus lateralis muscles^10^, and also more co-contraction of semitendinosus and vastus lateralis muscles^5^. In terms of kinematics and kinetics analyses during walking, a few studies compared hard sole and shaft boots with soft ones. Boots with a high and stiff shaft could decrease gait velocity, step length, ankle power generation by 33%^11^, and ankle and knee power absorption^5^. These alterations in gait biomechanics are speculated to compensate for the restricted ankle RoM and the reduced activation of muscles spanning the ankle joint^3,5^. In a recent study, Nesterovica-Petrikova et al. found that ankle RoM and angular velocity are affected in walking with boots compared to barefoot walking. However, inconsistencies exist among the results. For instance, Cikajlo and Matjacić found the ankle joint as the only affected site^11^, whereas Böhm and Hösl, and Kersting et al. observed compensations in the knee joint as a result of wearing hard boots^5,12^.

Lack of enough studies, along with conflicting results on the effects of boots on lower limb biomechanics, could lead to ambiguities in understanding the function of this specific type of footwear and their influence on our musculoskeletal system. Therefore, the aim of this study was to compare the influence of leather lace-up boots and casual shoes on kinematics and kinetics of lower limb joints during normal walking. We hypothesized that boots would alter the angle, moment, power, and work of the lower limb joints during walking, compared to casual shoes. The results of this study could provide valuable information for footwear design, selection, and prescription. They could also help to understand the biomechanical effects of boots on walking performance and injury risk. Furthermore, the findings may have implications for the clinical management of injuries associated with footwear in boot users. Finally, they could contribute to the development of more effective interventions that incorporate boots as part of the treatment or rehabilitation plan.

## Methods

### Study design

A cross-over, counter-balanced, within-subject comparison design was applied in this study, in which two footwear conditions in a counterbalanced order were tested on each participant in a single gait analysis session. The research protocol was approved locally by the university Biomedical Research Ethics Committee (IR.IAU.H.REC.1402.133), and all experiments were performed in accordance with relevant guidelines and regulations.

### Study participants

In total, twenty healthy young male university students with an average age of 23 ± 2.43 years, mass of 71.55 ± 10.47 kg, and height of 1.77 ± 0.08 m participated in this study. The required sample size was estimated based on the parameters of 80% power, 5% significance level and 0.72 effect size derived from ankle joint eccentric power reported by Böhm and Hösl^5^. The inclusion criteria were body mass index (BMI) less than 31 kg/m^2^, no history of any pain or orthopedics or neurological pathologies in lower limb that may influence walking, and being physically active. Each participant signed an informed consent prior to data collection, and they were not involved in the study design, analysis and interpretation of the findings.

### Footwear

**Figure 1:**
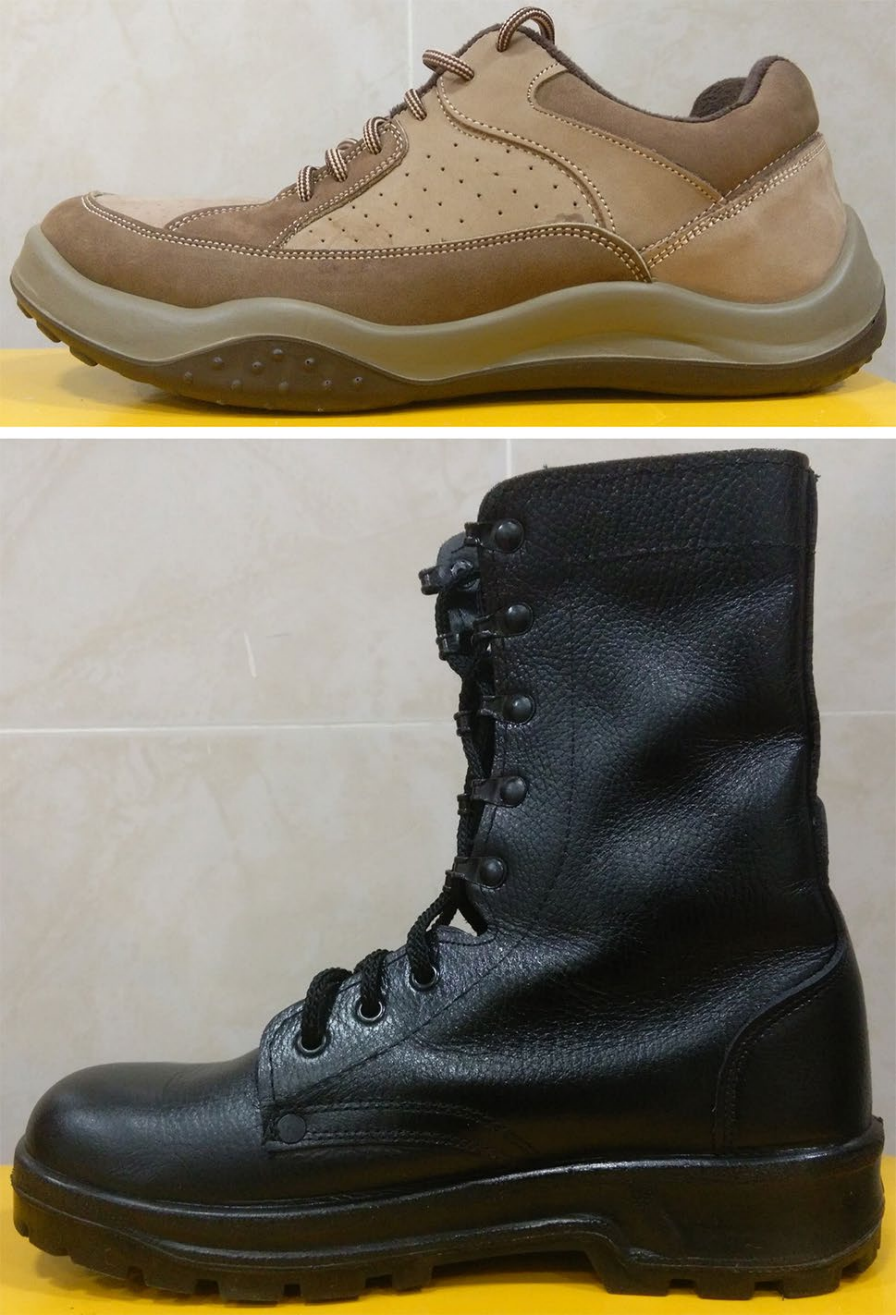
The footwear. The shoe (above) and the boot (below).

The two footwear conditions tested in this study, a boot and a casual shoe (Fig. 1), were provided locally in three European sizes ranging from 41 to 43. The shoes, which were made of Nubuck, were not as hard as the leather lace-up boots. Compared to the shoes, the boots had more mass (~ 600 vs. 1200 g), shaft height (12.5 cm), and heel-to-toe drop (5 vs. 20 cm). Also, the outsole of the shoes was Polyurethane with a hardness of 50 ShoreA and a density of 450 g/l, respectively. In contrast, the boots had a dual-density outsole made of the same Polyurethane as the shoes and Rubber with a hardness of 65 ShoreA and a density of 1150 g/l, increasing the stiffness and durability.

### Study procedures

Retro-reflective markers were attached on bony landmarks of C7 and the right and left acromion, superior iliac spines, knee epicondyles, ankle malleoli, calcaneus, 1st and 5th metatarsal heads. We also fixed a rigid plate with four non-collinear markers on the lateral side of the thigh and shank. Marker trajectories and ground reaction force (GRF) in 3D were measured using a motion capture system with eight infrared cameras (Miqus M3, Qualisys AB, Sweden) and a ground-embedded force plate (Kistler Instrumente AG, Switzerland) at sampling frequency of 200 Hz and 2 kHz, respectively. Both footwear conditions (the boot and the shoe) were tested on all participants. A static trial in a neutral standing posture and multiple self-paced walking trials on a 16-m walkway were recorded for every condition. The footwear conditions were tested in a counterbalanced order and participants had adequate time to warm up and rest before and during the trials.

### Data analysis

We analyzed 3–5 successful trials for each footwear condition (on average 8 trials for each participant). Heel strike events were detected using the foot vertical velocity algorithm^13^ and double-checked by visual inspection for all trials (since there was only one force plate). Hip joint center markers were created according to the modified Harrington regression equation with pelvis width only^14^. Knee and ankle joint centers were the average of medial and lateral markers of epicondyles and malleoli, respectively. Using the joint center markers for scaling thigh and shank segments were found to improve the estimation of the segments length compared to the corresponding MRI measures^15^.

A full-body constrained model^16^ with a six-degrees-of-freedom (DoF) joint between pelvis and ground, three rotational DoF lumbar and hip joints, one DoF knee joint with parameterized rotational and translational coordinates, and one DoF ankle and subtalar joints was employed in this study. Additionally, axes of rotation for the knee, ankle, and subtalar joints followed their physiological movement. The original model was adapted and simplified to fit our purpose by converting head, arms, and torso to a single rigid body, re-defining the metatarsophalangeal as a weld joint, changing the rotation sequence of pelvis to rotation, obliquity, and tilt^17^, removing the constraints over joints RoM, removing patella and its kinematics constraints, and removing all muscles, actuators, and wrapping geometries.

We scaled the generic model to fit each participant's anthropometry for each condition, based on joint centers and anatomical markers, followed by marker registration using the Scale tool in Opensim v.4.4^18^. The tool was executed twice in order to fine-tune the segments dimensions and markers positions. Additionally, the mass of the foot segment in the scaled model of the boot condition was adjusted to account for the footwear mass difference. Joint angles were computed from the experimental markers using the Kalman smoothing algorithm for inverse kinematics which was shown to be a robust algorithm in the presence of soft tissue artifacts^19^. Kinematics and GRF data were smoothed using a zero-phase low-pass Butterworth filter with a 14 Hz cut-off frequency. The Inverse Dynamics tool computed joint moments in the joint coordinate system as a clinically meaningful interpretation of net muscle activation^20^. We computed the knee adduction moment using the Joint Reaction tool as the external moment applied on the tibia and expressed in the tibia coordinate system^21^. Joint power was calculated as the product of joint moment (Nm) and joint angular velocity (rad/s)^22^. The net joint work was calculated as the time integration of the joint power, for both positive (concentric) and negative (eccentric) power phases, and for each muscle group^23^.

The time series of GRF in stance phase and joint angle, moment, and power in the entire gait cycle were time-normalized (0–100%) and averaged across each participant’s trials. Also, nondimentional quantities were computed and scaled to the normal participant^24^ using the average mass and leg length across all individuals (Eq. (1)):

1 $${S}_{force}=\frac{{m}_{norm}}{{m}_{i}}$$

$${S}_{moment}{=S}_{work}=\frac{{m}_{norm} . {l}_{norm}}{{m}_{i} . {l}_{i}}$$

$${S}_{power}=\frac{{m}_{norm} . \sqrt{{l}_{norm}}}{{m}_{i} . \sqrt{{l}_{i}}}$$

where $S$ is a scale factor computed for each participant according to their mass ${m}_{i}$ and leg length ${l}_{i}$, and the corresponding measures of normal mass ${m}_{norm}$ and leg length ${l}_{norm}$. Finally, the ensemble averages of joint kinematics and kinetics were low-pass filtered at 7 Hz.

### Statistical analysis

The D’Agostino-Pearson K^2^ test of normality was used to check the distribution of the data prior to statistical analysis. Discrete variables (walking velocity and joint work) as well as time-series data (GRF, Joint kinematics and kinetics) were compared between footwear conditions by paired-samples t-test. In the case of the nonparametric test^25^, we used 100 k permutations to achieve stable results numerically. Also, the results of both parametric and nonparametric tests agreed qualitatively. All statistical tests were two-sided with a significance level of P < 0.05 and conducted using the spm1d package^26^. Additionally, to see the clinical relevance of the differences between footwear conditions, effect size was calculated using Cohen’s d statistics, interpreted as 0.2 = small, 0.5 = medium, 0.8 = large, and 1.2 = very large^27^ and reported only for the significant differences. A custom-made script in Python v.3.8 was employed for all data and statistical processings.

## Results

**Figure 2:**
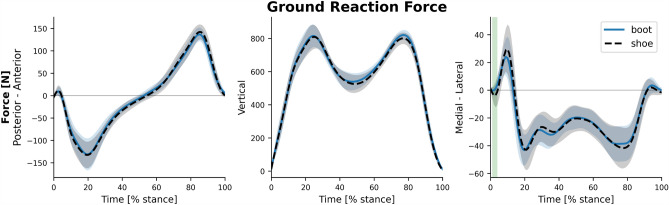
Ground reaction force. Depicted in anterior–posterior, vertical, and medial–lateral directions for the shoe (dashed black) and the boot (solid blue) conditions.

The results indicated no significant difference in walking velocity between conditions (shoe: 1.38 ± 0.12 m/s, boot: 1.36 ± 0.12 m/s, t(19) = 0.83, P = 0.41). The average ensemble graphs of GRF in three directions are presented in Fig. 2. There was only a significant difference between the footwear conditions in the mediolateral GRF in 1.8–3.5% of stance phase (P = 0.01, d = 0.73).

**Figure 3:**
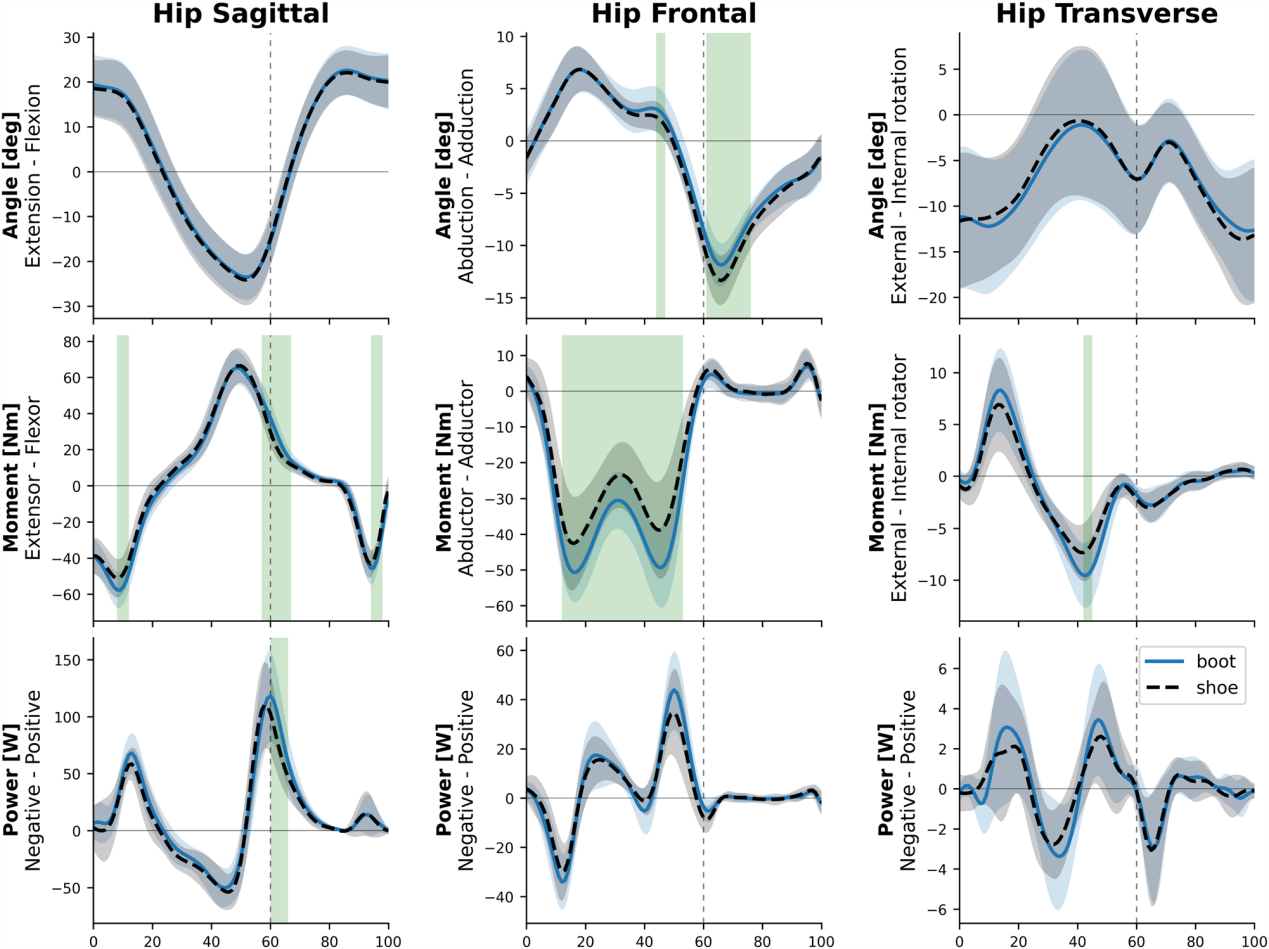
The kinematics and kinetics of the hip joint. Depicted in sagittal, frontal and transverse planes for the shoe (dashed black) and the boot (solid blue) conditions.

The ensemble averages of hip joint kinematics and kinetics in the sagittal, frontal and transverse planes throughout the entire gait cycle are presented in Fig. 3. In the sagittal plane, hip extensor moment in 8.3–11.9% (P = 0.009, d = 0.73) and 94.8–97.7% (P = 0.002, d = 0.99), hip flexor moment in 57.9–66% (P < 0.001, d = 1.11), and hip power generation in 60.7–66% (P < 0.001, d = 0.83) were significantly greater in boot condition. In the Frontal plane, the peak hip adduction angle in 44.4–46.9% (P = 0.02, d = 0.46) was greater in boot condition while the peak hip abduction angle in 61.7–75.4% (P = 0.003, d = 0.69) was lower in boot condition. The hip abductor moment in 12.2–52.3% (P < 0.001, d = 0.93) was significantly greater in boot condition. In the transverse plane, the peak hip external rotator in 42.8–44.3% (P = 0.04, d = 0.83) was significantly greater in boot condition.

**Figure 4:**
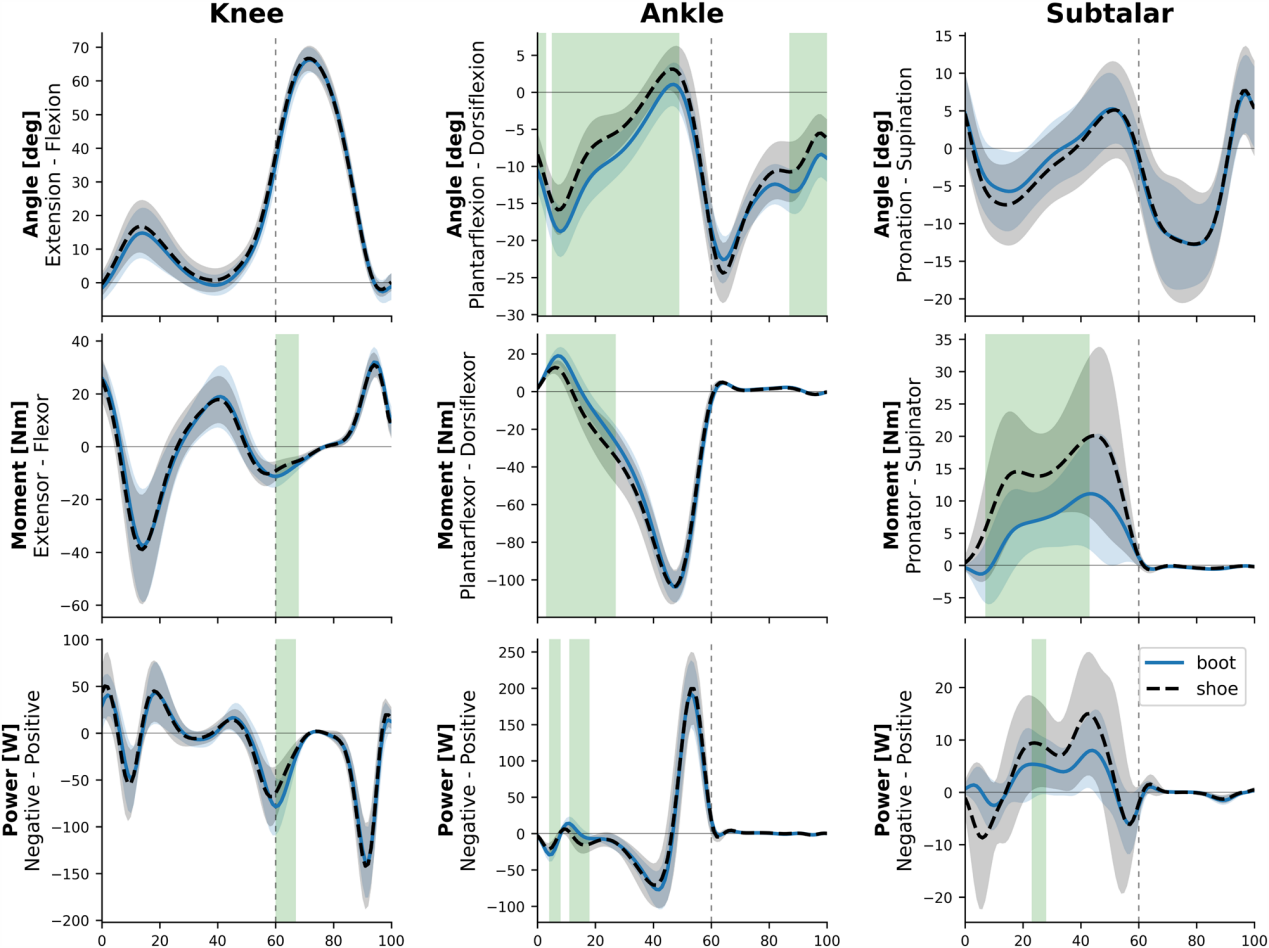
The kinematics and kinetics of the knee, ankle, and subtalar joints. Depicted for the shoe (dashed black) and the boot (solid blue) conditions.

**Figure 5:**
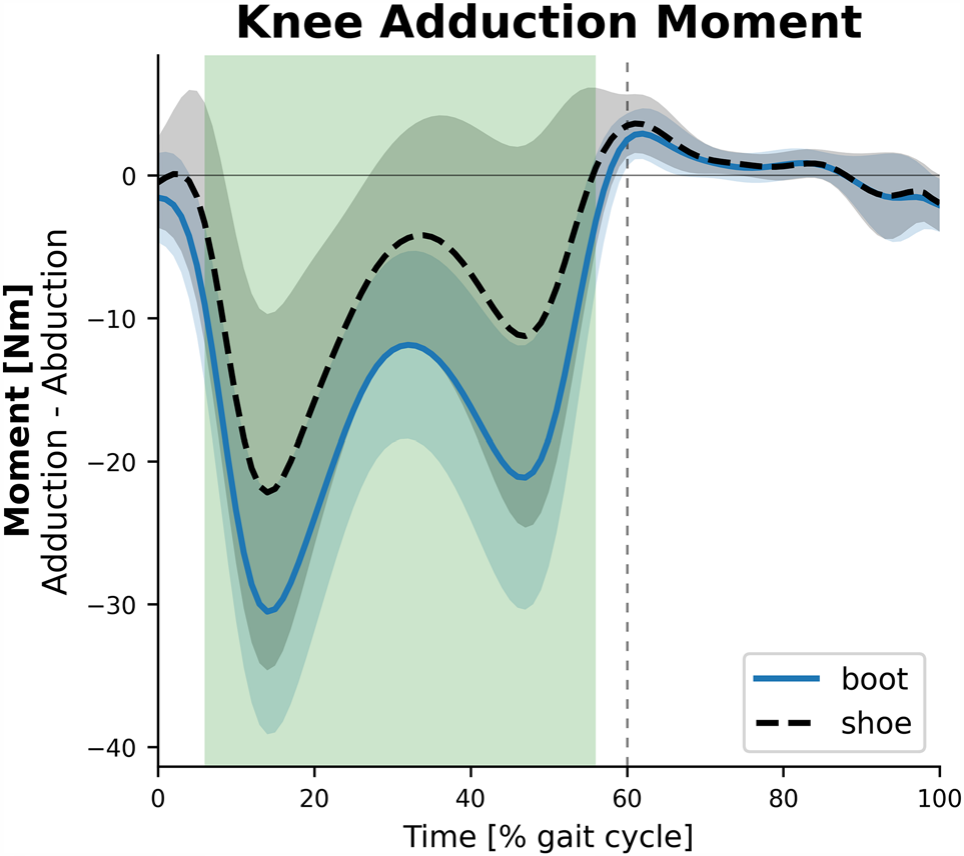
Knee adduction moment. Depicted for the shoe (dashed black) and the boot (solid blue) conditions.

**Figure 6:**
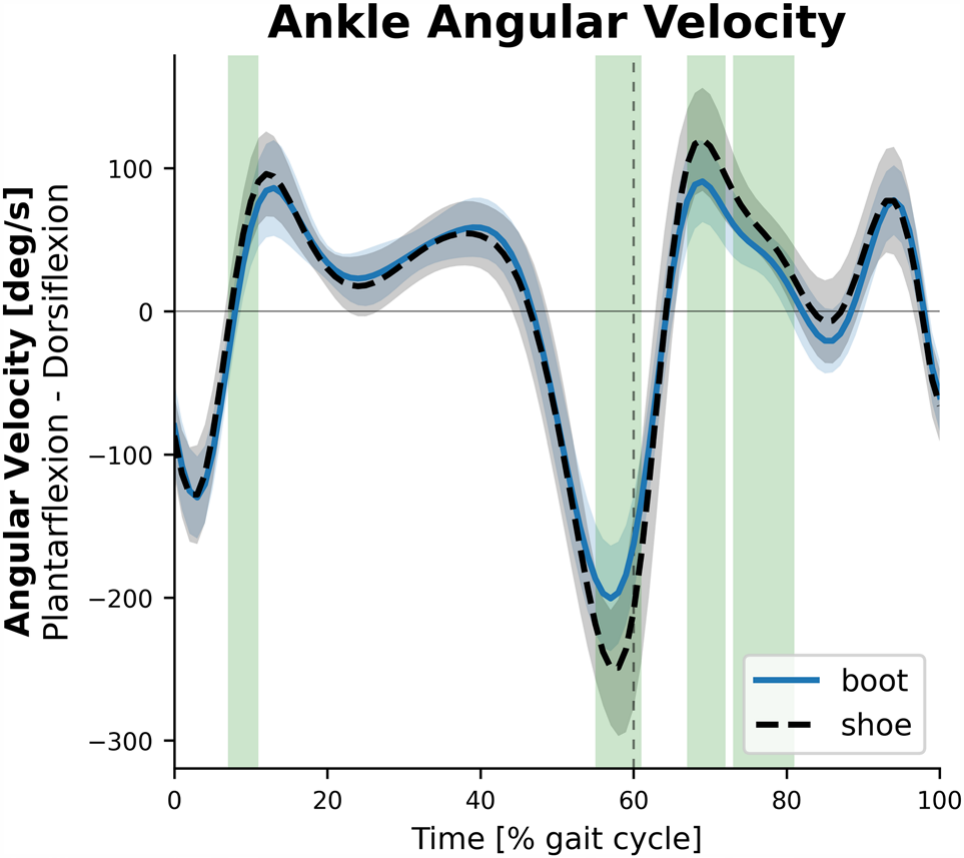
Ankle angular velocity. Depicted for the shoe (dashed black) and the boot (solid blue) conditions.

The average ensemble graphs of knee, ankle, and subtalar joints kinematics and kinetics in the entire gait cycle are presented in Fig. 4. The knee extensor moment in 60.2–67.3% (P < 0.001, d = 1.0), the peak knee power absorption in 60.8–66.2% (P < 0.001, d = 0.99), and the peak knee adduction moment in 6.6–55.7%, almost the entire stance phase, (P < 0.001, d = 1.09) were significantly greater in the boot condition (Fig. 5). The ankle was significantly more plantarflexed in the boot condition in almost the entire stance phase in 0–2.5% (P = 0.02, d = 0.76) and 5–48.1% (P < 0.001, d = 1.26), and the terminal swing in 87.4–100% (P = 0.001, d = 1.08). Significantly lower ankle dorsiflexion velocity in 7.2–10.4% (P = 0.005, d = 0.84) and 67.4–80.8% (P = 0.002, d = 0.95), as well as lower peak ankle plantarflexion velocity in 55.3–60.3% (P < 0.001, d = 1.28) was observed in the boot condition (Fig. 6). Also, the ankle dorsiflexor moment in 3.9–26.8% (P < 0.001, d = 1.59), ankle power absorption in 4.6–7.5% (P < 0.001, d = 1.1), and ankle power generation in 11.1–17.3% (P < 0.001, d = 1.25) were significantly greater in boot condition. The subtalar supinator moment in 7.9–42.6% (P < 0.001, d = 1.25) was significantly lower in boot condition.

**Figure 7:**
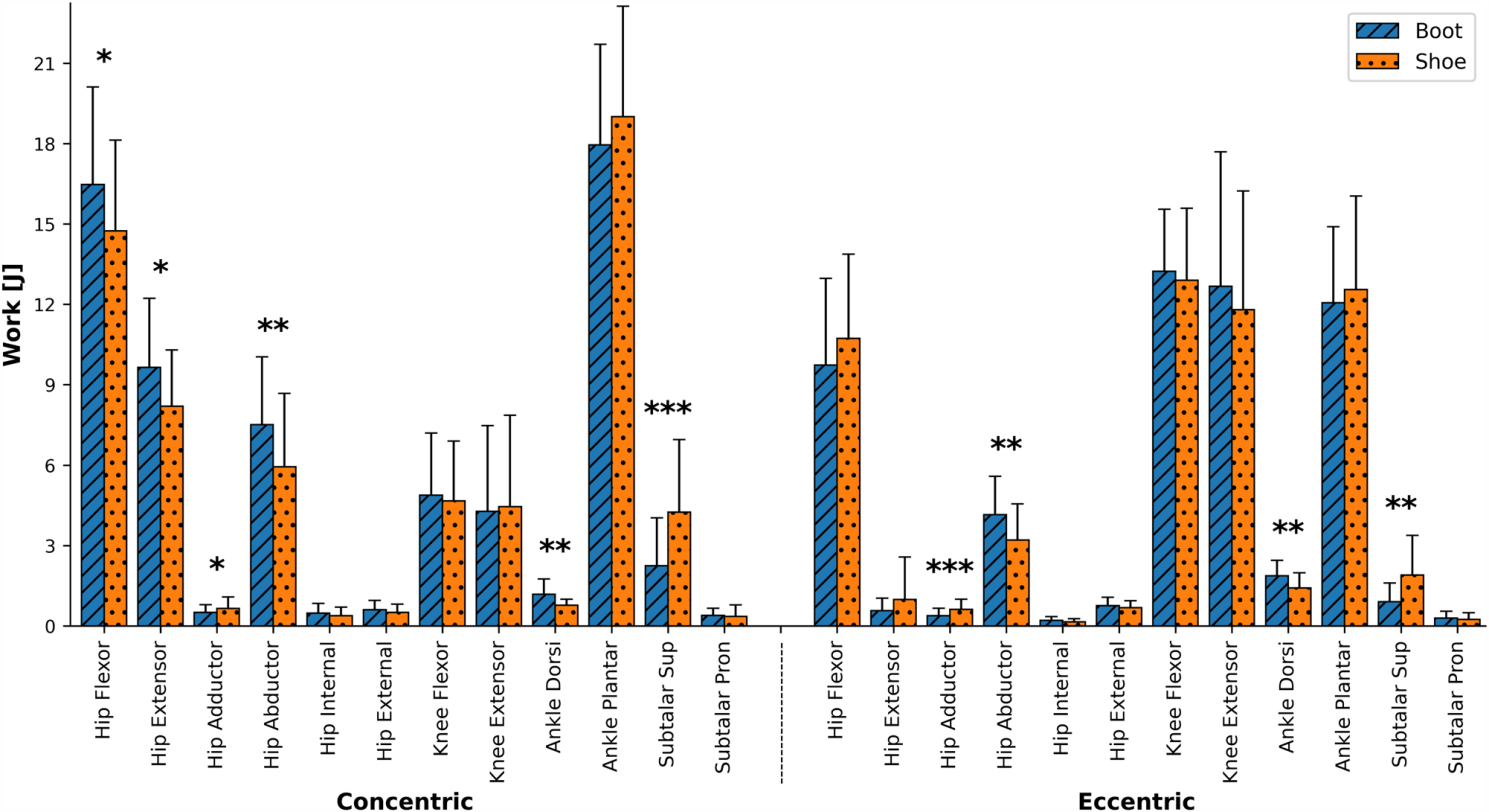
Joints work. Depicted in each muscle group for the shoe (dotted orange) and the boot (hashed blue) conditions.

The concentric and eccentric work for each muscle group of the lower limb joints are presented in Fig. 7. The boot condition increased the concentric work done by hip flexor (11.7%, P = 0.03, d = 0.5), hip extensor (17.7%, P = 0.02, d = 0.63), hip abductor (26.6%, P = 0.001, d = 0.62), ankle dorsiflexor (53.2%, P = 0.001, d = 0.98), while decreased it for hip adductor (-23%, P = 0.01, d = 0.44) and subtalar supinator (-47.2%, P < 0.001, d = 0.89) muscle groups. Also, the boot condition increased the eccentric work done by hip abductor (29.3%, P = 0.001, d = 0.7) and ankle dorsiflexor (31.7%, P = 0.001, d = 0.8), but decreased it for hip adductor (− 37.7%, P < 0.001, d = 0.7) and subtalar supinator (-52.6%, P = 0.004, d = 0.88) muscle groups. The net concentric work in boot condition was greater for the hip joint in sagittal (13.9%, P = 0.001, d = 0.78) and frontal (21.7%, P = 0.002, d = 0.56) planes and it was lower for subtalar joint (− 42.7%, P < 0.001, d = 0.9). The net eccentric work in boot condition was greater for hip joint in frontal (18.9%, P = 0.01, d = 0.51) and transverse (15.85%, P = 0.04, d = 0.42) planes and it was lower for the subtalar joint (− 44.6%, P = 0.004, d = 0.88).

## Discussion

This study aimed at investigating the influence of boots on lower limb joints kinematics and kinetics compared to casual shoes. To be concise, we only include studies in which the task of level walking has been investigated, because other tasks have different mechanics and are not related to our topic. Walking velocity was not statistically different between walking with boots and walking with casual shoes, so that it wouldn’t affect our gait variables. No significant differences in any percentage of stance phase were observed in any direction of GRF, except a small area at early stance in the mediolateral GRF which was significantly lower in the boot condition, whereas previous studies reported that walking with hard boots is associated with greater vertical GRF^4,28–30^. Although in our study the second peak of the vertical GRF in the boot condition was slightly greater than the shoe condition, this difference was not statistically significant. This could be probably due to the differences in footwear designs and materials among studies.

The most intuitive result in joint kinematics was the decreased ankle RoM in the entire gait cycle. Given that the boot has more heel-to-toe-drop compared to the shoe, the ankle joint was more plantarflexed at the heel strike as well as the rest of the stance phase as expected. These findings are consistent with the previous studies^4,5,11,12,31^. According to Hamill and Bensen (1996), wearing boots that restrict ankle dorsiflexion can impair agility performance by slowing down the reaction time^4^. The ankle dorsiflexor moment, power, and work in both eccentric and concentric contraction phases were significantly higher in the boot condition in the early stance phase of gait. This suggests that the ankle dorsiflexor muscles, particularly the Tibialis Anterior, are overactivated and overloaded, which is consistent with the EMG study of Schulze et al.^8^ and the muscle simulation study of Wright et al.^32^. This could increase the risk of disorders such as tibialis anterior muscle overuse injury, shin splint, and chronic exertional compartment syndrome^8,33^. Although the ankle plantarflexor works in both eccentric and concentric contraction phases of walking with boots were slightly less than those in the shoe condition, these differences were not statistically significant. Contrary to Böhm and Hösl^5^, and Kersting et al.^12^, the net ankle joint work was not different between the footwear conditions.

Unexpectedly, the subtalar joint kinematics was not affected by footwear condition, except a nonsignificant reduction in the range of pronation motion. That’s probably due to our definition of foot segment and the corresponding marker set (the application of forefoot markers to track the motion of the rearfoot segment and the subtalar joint). A one-segment foot model with forefoot markers as tracking markers likely fails to measure the subtalar joint kinematics and hence kinetics accurately^34^. We suggest implementing a multi-segment foot model or attaching more markers on the rearfoot for future studies. However, the boots significantly decreased the subtalar supinator moment and power generation in the stance phase, as well as the subtalar supinator concentric and eccentric works (Fig. 7), suggesting lower loads applied on the soft tissue in the medial side of the ankle. This may have clinical implications for disorders related to ligament and tendons such as tibialis posterior tendon dysfunction. More pathology-specific studies on the clinical population are required to better understand the clinical implications of boots.

The knee joint kinematics and kinetics (moment and power) in the stance phase were barely affected by the footwear conditions; this finding is consistent with Cikajlo and Matjacić^11^ and Böhm and Hösl^5^. Contrary to Böhm and Hösl and Kersting et al. who reported that the knee eccentric work in the stance phase was significantly increased during walking with stiff boots^5,12^, in our results, although the knee eccentric work in the entire gait cycle was slightly greater in the boot condition, this difference was not statistically significant.

The results demonstrated that there was no significant difference in the ankle plantarflexor moment and power generation at the time of push-off phase of gait between the boot and shoe conditions; this finding is consistent with Böhm and Hösl^5^, and in contrast with Cikajlo and Matjacić^11^. Further analysis on joint angular velocity (Fig. 6) also showed that, in accordance with Nesterovica-Petrikova et al.^31^, ankle plantarflexion velocity was notably reduced at the time of ankle push-off in the boot condition. On the other hand, there was significantly more hip flexor moment and power generation in the pre-swing and initial swing phases in the boot condition. These findings indicate that the moment generated by ankle plantarflexor muscles, which was equal in magnitude to the shoe condition, was not sufficient to produce sufficient joint velocity/acceleration, and thus, greater contribution of the hip joint to the propulsive power occurred^35^. In other words, hip joint pull-off strategy would compensate for the insufficient ankle joint push-off, and assist swing initiation and forward acceleration of the leg. The boot condition also increased the knee extensor moment and power absorption in the initial swing phase of gait. This could indicate that the more net propulsive power generated by ankle push-off and hip pull-off strategies in the boot condition requires more eccentric contraction of knee extensors (power absorption) to help smooth the gait^35^. This is in agreement with the EMG study in which greater activation of rectus femoris was observed during walking with stiff boots^8^. This bi-articular muscle functions as the main contributor to both hip joint power generation and knee joint power absorption in pre- and early swing phases of gait^22^. Increased activation of rectus femoris, as well as other quadriceps muscles which was found in EMG studies to be greater in hard boots^9,10^, could increase the compressive force on the patella and cause pain. Accordingly, Sinclar et al. found that patellofemoral contact force, loading rate, and pressure were significantly greater when running with military boots compared to running shoes^36^. A temporal shift in the power generation (concentric) phase of hip flexors as well as the power absorption (eccentric) phase of knee extensors is evident in the graphs, highlighting the compensatory adjustments at hip and knee joints in response to the reduced ankle RoM and angular velocity as well as the insufficient ankle power generation. We speculate that several boot features such as the shaft height, shaft stiffness, sole stiffness, heel-to-toe-drop, and mass are the causes of such biomechanical alterations in the propulsion phase of gait.

Further analysis on the knee joint moment revealed that external adduction moment was significantly greater in the boot condition in almost the entire stance phase of gait (Fig. 5). During normal walking, the ground reaction vector passes medially to the knee joint center, generating an adduction moment that tends to adduct the knee joint. So, the majority of the loads are applied to the medial compartment of the knee^37^ that could be associated with degenerative effects on the cartilage tissue and joint space narrowing over time. There is a strong relationship between the knee adduction moment and joint loading^38^ and therefore, the onset, severity, and progression of osteoarthritis in the medial compartment of the knee^39,40^. Furthermore, excessive external knee adduction moment exerts abnormal load on the soft tissues on the lateral side of the knee such as knee abductor muscles, ligaments, and joint capsule, causing rupture^21^ and joint instability. It is also a predictor of ACL injury and patellofemoral pain in landing tasks^41,42^. Consequently, this may lead to pain, functional impairment and disability.

Hip joint angles in sagittal and transverse planes were not statistically different between footwear conditions. In the frontal plane, however, the hip joint adduction angle was significantly greater in the boot condition in a small area in late-stance, followed by a significantly lower abduction angle in the initial swing phase. The hip extensor moment in the loading response phase was significantly greater in boot condition, highlighting a greater need to control the forward acceleration of the trunk and hip. This was previously found to be a compensation strategy in lower limb prosthesis users^35,43^. In the terminal swing phase, the hip extensor moment was also significantly greater in the boot condition, indicating a greater need for hip extensor muscles to decelerate the forward swinging leg^35^. This might be as a result of the greater boot mass that increases the moment of inertia of the leg, and hence, inertial forces and moments used in Inverse Dynamics calculations^44^. The greater hip external rotator and abductor moments and concentric and eccentric works observed in the boot condition could imply that more pelvic stability^35^ is required during walking with boots. Contrary to Böhm and Hösl and Kersting et al. who didn’t observe any significant differences in the net concentric and eccentric works at the hip joint^5,12^, in our results, they were significantly greater in the boot condition. Overall, the significantly increased hip extensor, abductor, and external rotator moments and the net hip joint work in the boot condition suggest that walking with boots may increase the muscles activation, force, and hence, the hip joint contact force which might be a risk factor for developing hip joint degenerative diseases over time. Previous research has established a link between the cumulative moment of the hip joint, notably in the frontal plane, and the subsequent advancement of radiographic signs of hip osteoarthritis^45^.

Based on the findings of the present study, compensatory changes in response to the reduced ankle RoM and angular velocity occurred at not only the knee joint, but also at the hip joint and the later one potentially contributes to the propulsive power of gait required for an efficient swing phase. Such adaptations were previously seen in individuals who used ankle foot orthosis or transtibial prosthesis which compromise ankle power generation during push-off phase of gait^35,43^. To the best of the author's knowledge, this is the first study that addresses such alteration in lower limb joints biomechanics during walking with boots compared to casual shoes. Further studies are suggested in this field to confirm the present findings that overall would provide useful information for designing footwear and developing more effective interventions for boot-related injuries or conditions that require boots as part of the treatment or rehabilitation plan. Moreover, according to the present findings, it may be suggested that wearing boots could be effective for the prevention and management of disorders related to ankle ligament and tendons. More analysis of cross-correlation or coordination between joints kinematics and kinetics are suggested to better explain these results.

Our gait modeling, which implemented constrained inverse kinematics, physiological movement of the knee joint, a robust regression equation for hip joint center estimation, and Kalman Smoothing algorithm, would enhance the accuracy of our simulation compared to the conventional gait model^46^ used in the previous studies^5,11^. This approach, in addition to footwear differences, may explain the conflicting results between our findings and the previous studies to some extent. This study was limited by the lack of data on muscle activation and strength. Furthermore, we could not apply any algorithms to reduce residual forces and moments accounted for dynamic inconsistencies^47^, as there was only one force plate and we were not able to measure all the external forces on the body in a gait cycle. In this study, only the healthy young men were included, which makes it difficult to generalize the results to women or other age groups. We suggest further analyses on muscle force and joint load to better understand the alterations and adaptations in response to walking with boots. More prospective cohort studies are also required to understand the risk factors for boot-related injuries in the long-term.

## Conclusion

This study found that more contribution of the hip joint to the propulsive power of gait is required during walking with boots compared to casual shoes. Reduced ankle RoM and angular velocity, as well as insufficient ankle power generation in gait lead to compensatory adjustments at hip and knee joints that may apply excessive and abnormal loads on hip and knee joints and muscles. Consequently, this may increase the likelihood of experiencing musculoskeletal injuries and osteoarthritis. It is imperative to undertake a comprehensive overhaul of the design of boots with the aim of mitigating their adverse effects. However, lower loads on the soft tissue in the medial side of the ankle as the result of wearing boots may have clinical implications for disorders related to ankle joint ligament and tendons.

## Data Availability

The datasets relevant to the current study are available from the corresponding author on reasonable request.

## References

[1] Williams, A. E. & Nester, C. *Pocket Podiatry: Footwear and Foot Orthoses E-Book*. (Elsevier Health Sciences, UK, 2010).

[2] Hsu, J. D., Michael, J. & Fisk, J. *AAOS Atlas of orthoses and assistive devices e-book*. (Elsevier Health Sciences, UK, 2008).

[3] Dobson, J. A., Riddiford-Harland, D. L., Bell, A. F. & Steele, J. R. Work boot design affects the way workers walk: A systematic review of the literature. *Appl. Ergon.* **61**, 53–68 (2017). 28237020 10.1016/j.apergo.2017.01.003

[4] Hamill, J. & Bensel, C. K. *Biomechanical analysis of military boots. Phase II: Human user testing of military and commercial footwear*. (U.S. Army Natick Research, Development and Engineering Center, UK, 1996).

[5] Böhm, H. & Hösl, M. Effect of boot shaft stiffness on stability joint energy and muscular co-contraction during walking on uneven surface. *J. Biomech.* **43**, 2467–2472 (2010). 20541206 10.1016/j.jbiomech.2010.05.029

[6] Orr, R. *et al.* The impact of footwear on occupational task performance and musculoskeletal injury risk: A scoping review to inform tactical footwear. *Int. J. Environ. Res. Public. Health* **19**, 10703 (2022). 36078419 10.3390/ijerph191710703 PMC9518076

[7] Chiou, S. S., Turner, N., Zwiener, J., Weaver, D. L. & Haskell, W. E. Effect of boot weight and sole flexibility on gait and physiological responses of firefighters in stepping over obstacles. *Hum. Factors J. Hum. Factors Ergon. Soc.* **54**, 373–86 (2012). 10.1177/0018720811433464 22768640

[8] Schulze, C. *et al.* The influence in airforce soldiers through wearing certain types of army-issue footwear on muscle activity in the lower extremities. *Open Orthop. J.* **5**, 302–306 (2011). 21886685 10.2174/1874325001105010302 PMC3162209

[9] Kim, M.-K., Kim, Y.-H. & Yoo, K.-T. Effects of shoe type on lower extremity muscle activity during treadmill walking. *J. Phys. Ther. Sci.* **27**, 3833–3836 (2015). 26834363 10.1589/jpts.27.3833 PMC4713802

[10] Dobson, J. A., Riddiford-Harland, D. L. & Steele, J. R. Effects of wearing gumboots and leather lace-up boots on lower limb muscle activity when walking on simulated underground coal mine surfaces. *Appl. Ergon.* **49**, 34–40 (2015). 25766420 10.1016/j.apergo.2015.01.006

[11] Cikajlo, I. & Matjačić, Z. The influence of boot stiffness on gait kinematics and kinetics during stance phase. *Ergonomics* **50**, 2171–2182 (2007). 17886013 10.1080/00140130701582104

[12] Kersting, U. G., Støttrup, N. & Larsen, F. G. The influence of shaft stiffness on joint kinematics and kinetics during hiking. *J. Biomech.* **126**, 110643 (2021). 34358901 10.1016/j.jbiomech.2021.110643

[13] O’Connor, C. M., Thorpe, S. K., O’Malley, M. J. & Vaughan, C. L. Automatic detection of gait events using kinematic data. *Gait Posture* **25**, 469–474 (2007). 16876414 10.1016/j.gaitpost.2006.05.016

[14] Sangeux, M. On the implementation of predictive methods to locate the hip joint centres. *Gait Posture* **42**, 402–405 (2015). 26215642 10.1016/j.gaitpost.2015.07.004

[15] Kainz, H. *et al.* Accuracy and reliability of marker-based approaches to scale the pelvis, thigh, and shank segments in musculoskeletal models. *J. Appl. Biomech.* **33**, 354–360 (2017). 28290736 10.1123/jab.2016-0282

[16] Lai, A. K. M., Arnold, A. S. & Wakeling, J. M. Why are antagonist muscles co-activated in my simulation? A musculoskeletal model for analysing human locomotor tasks. *Ann. Biomed. Eng.* **45**, 2762–2774 (2017). 28900782 10.1007/s10439-017-1920-7 PMC5989715

[17] Baker, R. Pelvic angles: A mathematically rigorous definition which is consistent with a conventional clinical understanding of the terms. *Gait Posture* **13**, 1–6 (2001). 11166548 10.1016/S0966-6362(00)00083-7

[18] Seth, A. *et al.* OpenSim: Simulating musculoskeletal dynamics and neuromuscular control to study human and animal movement. *PLoS Comput. Biol.* **14**, e1006223 (2018). 30048444 10.1371/journal.pcbi.1006223 PMC6061994

[19] De Groote, F., De Laet, T., Jonkers, I. & De Schutter, J. Kalman smoothing improves the estimation of joint kinematics and kinetics in marker-based human gait analysis. *J. Biomech.* **41**, 3390–3398 (2008). 19026414 10.1016/j.jbiomech.2008.09.035

[20] Schache, A. G. & Baker, R. On the expression of joint moments during gait. *Gait Posture* **25**, 440–452 (2007). 17011192 10.1016/j.gaitpost.2006.05.018

[21] Derrick, T. R. *et al.* ISB recommendations on the reporting of intersegmental forces and moments during human motion analysis. *J. Biomech.* **99**, 109533 (2020). 31791632 10.1016/j.jbiomech.2019.109533

[22] Winter, D. A. *Muscle Anthropometry* (Wiley, 2009).

[23] Wretenberg, P. & Arborelius, U. P. Power and work produced in different leg muscle groups when rising from a chair. *Eur. J. Appl. Physiol.* **68**, 413–417 (1994). 10.1007/BF00843738 8076621

[24] Hof, A. L. Scaling and normalization. *Handb. Hum. Motion N. Y. Springer* 295–305 (2018).

[25] Nichols, T. E. & Holmes, A. P. Nonparametric permutation tests for functional neuroimaging: A primer with examples. *Hum. Brain Mapp.* **15**, 1–25 (2002). 11747097 10.1002/hbm.1058 PMC6871862

[26] Pataky, T. C. One-dimensional statistical parametric mapping in Python. *Comput. Methods Biomech. Biomed. Engin.* **15**, 295–301 (2012). 21756121 10.1080/10255842.2010.527837

[27] Sawilowsky, S. S. New effect size rules of thumb. *J. Mod. Appl. Stat. Methods* **8**, 597–599 (2009). 10.22237/jmasm/1257035100

[28] Bini, R. R., Kilpp, D. D., Júnior, P. A. & Muniz, A. M. Comparison of Ground Reaction Forces between Combat Boots and Sports Shoes. *Biomechanics* **1**, 281–289 (2021). 10.3390/biomechanics1030023

[29] Lin, C.-L., Wang, M.-J.J. & Drury, C. G. Biomechanical, physiological and psychophysical evaluations of clean room boots. *Ergonomics* **50**, 481–496 (2007). 17575710 10.1080/00140130600901579

[30] Mara de Souza Muniz, A. *et al.* Effects from loaded walking with polyurethane and styrene-butadiene rubber midsole military boots on kinematics and external forces: A statistical parametric mapping analysis. *Appl. Ergon.* **94**, 103429 (2021). 10.1016/j.apergo.2021.103429 33862308

[31] Nesterovica - Petrikova, D., Vaivads, N. & Stepens, A. Effects of Tactical Boots on Foot and Ankle Kinematics. In: Dekhtyar, Y. & Saknite, I. (eds) *19th Nordic-Baltic Conference on Biomedical Engineering and Medical Physics*. Vol. 89 (Springer Nature Switzerland, UK, 2023). 112–118

[32] Wright, I. C., Neptune, R. R., van Den Bogert, A. J. & Nigg, B. M. Passive regulation of impact forces in heel-toe running. *Clin. Biomech.* **13**, 521–531 (1998). 10.1016/S0268-0033(98)00025-4 11415830

[33] Miller, S. *Mechanical factors affecting the estimation of tibialis anterior force using an EMG-driven modelling approach* (Brunel University, 2015).

[34] Pothrat, C., Authier, G., Viehweger, E., Berton, E. & Rao, G. One-and multi-segment foot models lead to opposite results on ankle joint kinematics during gait: Implications for clinical assessment. *Clin. Biomech.* **30**, 493–499 (2015). 10.1016/j.clinbiomech.2015.03.004 25812728

[35] Sloot, L. H. & Van der Krogt, M. M. Interpreting joint moments and powers in gait. In: Sloot, L. H (eds) *Handbook of human motion* (Springer International Publishing AG, 2017). 625–43

[36] Sinclair, J., Taylor, P. J. & Atkins, S. Effects of new military footwear on knee loading during running. *Footwear Sci.* **7**, 165–171 (2015). 10.1080/19424280.2015.1066879

[37] Shelburne, K. B., Torry, M. R., Steadman, J. R. & Pandy, M. G. Effects of foot orthoses and valgus bracing on the knee adduction moment and medial joint load during gait. *Clin. Biomech.* **23**, 814–821 (2008). 10.1016/j.clinbiomech.2008.02.005 18362043

[38] Manal, K., Gardinier, E., Buchanan, T. S. & Snyder-Mackler, L. A more informed evaluation of medial compartment loading: The combined use of the knee adduction and flexor moments. *Osteoarthritis Cartilage* **23**, 1107–1111 (2015). 25862486 10.1016/j.joca.2015.02.779 PMC4470852

[39] Sharma, L. *et al.* Knee adduction moment, serum hyaluronan level, and disease severity in medial tibiofemoral osteoarthritis. *Arthritis Rheum.* **41**, 1233–1240 (1998). 9663481 10.1002/1529-0131(199807)41:7<1233::AID-ART14>3.0.CO;2-L

[40] Hurwitz, D. E., Ryals, A. B., Case, J. P., Block, J. A. & Andriacchi, T. P. The knee adduction moment during gait in subjects with knee osteoarthritis is more closely correlated with static alignment than radiographic disease severity, toe out angle and pain. *J. Orthop. Res.* **20**, 101–107 (2002). 11853076 10.1016/S0736-0266(01)00081-X

[41] Hewett, T. E. *et al.* Biomechanical measures of neuromuscular control and valgus loading of the knee predict anterior cruciate ligament injury risk in female athletes: A prospective study. *Am. J. Sports Med.* **33**, 492–501 (2005). 15722287 10.1177/0363546504269591

[42] Myer, G. D. *et al.* High knee abduction moments are common risk factors for patellofemoral pain (PFP) and anterior cruciate ligament (ACL) injury in girls: Is PFP itself a predictor for subsequent ACL injury?. *Br. J. Sports Med.* **49**, 118–122 (2015). 24687011 10.1136/bjsports-2013-092536 PMC4182160

[43] Winter, D. A. & Sienko, S. E. Biomechanics of below-knee amputee gait. *J. Biomech.* **21**, 361–367 (1988). 3417688 10.1016/0021-9290(88)90142-X

[44] Richards, J. 2018. Inverse Dynamics theory. In: Richards, J. (eds) *The Comprehensive Textbook of Clinical Biomechanics with access to e-learning course [formerly Biomechanics in Clinic and Research]* (Elsevier Health Sciences, UK). 124–36

[45] Tateuchi, H. *et al.* Daily cumulative hip load is associated with radiographic progression of secondary hip osteoarthritis. *Osteoarthritis Cartilage* **25**, S21 (2017). 10.1016/j.joca.2017.02.049 28232145

[46] Baker, R., Leboeuf, F., Reay, J. & Sangeux, M. The Conventional Gait Model - Success and Limitations. In *Handbook of Human Motion* (eds Müller, B. *et al.*) 489–508 (Springer International Publishing, 2017).

[47] Hicks, J. L., Uchida, T. K., Seth, A., Rajagopal, A. & Delp, S. L. Is my model good enough? Best practices for verification and validation of musculoskeletal models and simulations of movement. *J. Biomech. Eng.* **137**, 020905 (2015). 25474098 10.1115/1.4029304 PMC4321112

